# Transcriptome Analysis of Redox Systems and Polyamine Metabolic Pathway in Hepatoma and Non-Tumor Hepatocyte-like Cells

**DOI:** 10.3390/biom13040714

**Published:** 2023-04-21

**Authors:** Olga N. Ivanova, George S. Krasnov, Anastasiya V. Snezhkina, Anna V. Kudryavtseva, Vyacheslav S. Fedorov, Natalia F. Zakirova, Michail V. Golikov, Sergey N. Kochetkov, Birke Bartosch, Vladimir T. Valuev-Elliston, Alexander V. Ivanov

**Affiliations:** 1 Engelhardt Institute of Molecular Biology, Russian Academy of Sciences, 119991 Moscow, Russia; 2 Lyon Cancer Research Center, Université Claude Bernard Lyon 1, INSERM U1052, CNRS 5286, 69008 Lyon, France

**Keywords:** polyamines, reactive oxygen species, antioxidant enzymes, urea cycle, proline metabolism, HepaRG

## Abstract

Reactive oxygen species (ROS) play a major role in the regulation of various processes in the cell. The increase in their production is a factor contributing to the development of numerous pathologies, including inflammation, fibrosis, and cancer. Accordingly, the study of ROS production and neutralization, as well as redox-dependent processes and the post-translational modifications of proteins, is warranted. Here, we present a transcriptomic analysis of the gene expression of various redox systems and related metabolic processes, such as polyamine and proline metabolism and the urea cycle in Huh7.5 hepatoma cells and the HepaRG liver progenitor cell line, that are widely used in hepatitis research. In addition, changes in response to the activation of polyamine catabolism that contribute to oxidative stress were studied. In particular, differences in the gene expression of various ROS-producing and ROS-neutralizing proteins, the enzymes of polyamine metabolisms and proline and urea cycles, as well as calcium ion transporters between cell lines, are shown. The data obtained are important for understanding the redox biology of viral hepatitis and elucidating the influence of the laboratory models used.

## 1. Introduction

The study of the functioning of redox systems in health and disease is one of the most dynamically developing areas of biochemistry and molecular biology. In recent years, much evidence has been obtained demonstrating that reactive oxygen species (ROS) and the redox-dependent post-translational modifications of proteins are involved in the regulation of signaling cascades, metabolic processes, and, as a result, of cell growth and differentiation [1,2,3]. Altered ROS production and neutralization affect cell viability, can cause (de)differentiation, and, thus, contribute significantly to the development of a wide range of pathologies [2]. Therefore, the investigation of the redox-dependent systems of a cell is an important direction for molecular biology.

The results of such studies strongly depend on the choice of adequate physiological models. In liver research, most studies are performed on hepatocarcinoma lines (Huh7, Huh7.5, HepG2, Hep3B, etc.) [4,5], although it is accepted that neoplastic transformation alters metabolism to ensure the rapid growth of tumors [6]. Metabolic processes are closely connected with the redox status of the cell: the catabolism of various substances is accompanied by the production of ROS and the oxidation of NAD(P)H to NAD(P), while biosynthesis processes, on the other hand, often require reduced NAD(P)H [7]. In contrast, non-transformed hepatocytes have a very low growth rate. The use of primary human hepatocytes (PHH) is often impossible due to the lack of donor material, difficult isolation procedures, and the high heterogeneity of the health status and genetic background of donors. Moreover, PHHs do not proliferate and can only be cultivated for a short time due to low viability (they die within 1 to 3 weeks). An alternative to PHHs is the HepaRG liver progenitor cell line, which can be differentiated into a co-culture of hepatocyte- and cholangiocyte-like cells [8]. Differentiated HepaRG cells can live without division for many weeks [9]. Importantly, the levels of expression of many hepatocyte-specific genes in these cells are much higher than in hepatocarcinoma cell lines, reaching the levels observed in PHHs [9,10]. Therefore, the HepaRG cell line has become a valuable tool for studies of the toxicology/metabolism of compounds and for investigations into the replication of hepatitis B and delta viruses [9,11]. Unfortunately, there are no data on the expression status of redox and metabolic enzymes. So, the first goal of this study was to analyze the differences in expression of ROS-producing enzymes, which are proteins that protect the cell from oxidative stress and maintain calcium homeostasis.

**Scheme 1:**
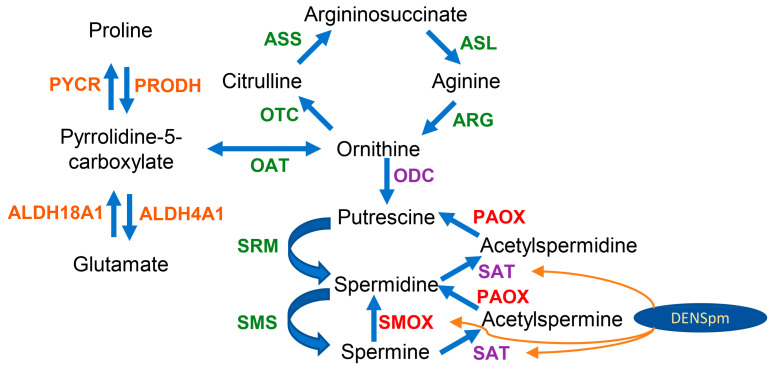
Metabolism of biogenic polyamines and its connection with the urea cycle and proline- and glutamate-metabolizing pathways. The enzymes are given in green, red, purple, or orange. Polyamine oxidases SMOX and PAOX (red) generate hydrogen peroxide. Ornithine decarboxylase (ODC) and spermidine/spermine-N^1^-acetyltransferases (SAT) (purple) are regulated by the antioxidant Nrf2 transcription factor. The enzymes given in orange are NAD(P)/NAD(P)H or FADH2-dependent, linking their activity to cellular redox status.

Biogenic polyamines are positively charged aliphatic oligocations that play an important role in the regulation of redox status and cell differentiation [12,13]. These include spermine, spermidine, and a number of other compounds across different species. Spermine and spermidine are present in all cell types at millimolar and submillimolar concentrations, which are thoroughly maintained by several enzymes [13]. The key regulated proteins of polyamine biosynthesis are ornithine decarboxylase (ODC) and S-adenosine methionine decarboxylase (AdoMetDC, AMD) and those of catabolism are spermidine/spermine N^1^-acetyltransferase (SSAT) and spermine oxidase (SMO, SMOX) (Scheme 1) [12,13]. Their altered expression induces changes in the levels of spermine and spermidine, contributing to the development of various diseases. Polyamine levels are elevated in many types of tumors, so one of the strategies for the development of antitumor drugs is to decrease the levels of spermine/spermidine by inhibiting their biosynthesis or the activation of catabolism [14,15].

The metabolism of polyamines is closely connected with ROS production. First, the expression of ODC and SSAT is regulated by the redox-dependent transcription factor Nrf2 (Scheme 1) [16,17]. ODC is also controlled by two alternative degradation pathways, one of which involves interaction with the antioxidant enzyme NAD(P)H quinone oxidoreductase 1 (Nqo1) [18]. Second, polyamine catabolism is associated with the production of hydrogen peroxide and toxic 3-aminopropanal, which is converted into acrolein [19,20]. Third, several papers demonstrated that polyamines can both act as ROS scavengers and regulate the expression of various antioxidant proteins [12]. However, there are almost no data on the effect of alteration of polyamine metabolism in non-tumor cells on the expression of ROS-producing and ROS-neutralizing genes, as well as that of the genes of linked metabolic pathways. So, the second goal was to analyze changes in the redox enzyme expression during the activation of polyamine catabolism in HepaRG cells.

Previously we showed that the activation of polyamine catabolism by treatment with N^1^,N^11^-diethyl-nor-spermine (DENSpm) induces dedifferentiation that resembles epithelial-mesenchymal transition [21]. However, despite the transcriptomic analysis performed in that paper, we did not assess the differences in the transcription levels of redox enzymes and regulatory proteins or the enzymes of polyamine and related metabolic systems. So, the current study fills this gap through the targeted re-analysis of that RNAseq dataset.

## 2. Materials and Methods

Fetal bovine serum (FBS), William’s medium, dimethyl sulfoxide (DMSO), bovine insulin, and hydrocortisone hemisulfate were obtained from Sigma (St. Louis, MO, USA). DMEM medium was obtained from Gibco (Carlsbad, CA, USA). Primary murine antibodies to proline dehydrogenase (PRODH, sc-376401) and secondary with horse radish peroxidase (HRP)-conjugated antibodies to murine IgG (sc-2005) were obtained from Santa-Cruz Biotechnologies (Santa-Cruz, CA, USA), while primary antibodies to NAD(P)H:quinone oxidoreductase (Nqo1, ab28947) and argininosuccinate syntase (ASS, ab124465) were from Abcam (Cambridge, UK). DENSpm was synthesized according to tahe previously described method [21]. The Huh7.5 cell line was a kind gift from Prof. Charles Rice (Rockefeller University, New York, NY, USA) and Apath L.L.C (St. Louis, MO, USA). The HepaRG cell line was previously described in [22].

### 2.1. Cell Culture

Huh7.5 cells were maintained in DMEM supplemented with 10% FBS and 2 mM glutamine and harvested at 80–90% confluency. The HepaRG cell line was cultured in Williams medium supplemented with 10% fetal bovine serum, 5 μg/mL insulin, 50 μM hydrocortisone, 50 U/mL penicillin, and 50 μg/mL streptomycin. For differentiation, the HepaRG cells were maintained without division in a 100% monolayer for 2 weeks in the complete William’s medium, and then for another 2 weeks in the complete William’s medium supplemented with 1.8% DMSO. Throughout the experiment, the medium was changed every three days. Treatment with 5 μM DENSpm was performed for 3 days. All cells were harvested by scraping and stored at −80°C for analysis. All cells were grown and harvested in triplicates.

### 2.2. Purification of RNA for NextSeq Sequencing

Total RNA was purified with the MagNA Pure Compact RNA Isolation Kit (Roche, Switzerland) on a MagNA Pure Compact Instrument (Roche). The quality of the isolated RNA was checked using Agilent 2100 Bioanalyzer (Agilent Technologies, Santa-Clara, CA, USA). Only RNA with RIN values (Integral RNA Integrity Level) of 8 or above was used in this research. RNA concentration was measured using a Qubit 2.0 Fluorometer (Thermo Fisher Scientific, Waltham, MA, USA).

### 2.3. Preparation and Transcriptome Sequencing of cDNA Library

The cDNA library was prepared using the TruSeq RNA Library Preparation Kit v2 (Illumina, San-Diego, CA, USA) according to the vendor’s protocol. mRNA was isolated using magnetic beads with (dT)-oligomers. After isolation, the mRNA was fragmented and then converted into double-stranded cDNA that was ligated with adapters and amplified. The quality of the final cDNA libraries was assessed using quantitative PCR reaction on a Rotor-Gene Q Instrument (Qiagen, Hinden, Germany). The sequencing was performed as described previously [21]. The raw FASTQ Illumina sequence data has been deposited to the Sequence Reading Archive (SRA) (accession number PRJNA494568).

### 2.4. Bioinformatic Analysis

Bioinformatic analysis has been described in [21]. Briefly, reads were trimmed, filtered, and mapped to the reference human genome hg38 (Ensembl release 97) with STAR [23]. The 3′-bias was evaluated with RSeQC [24]. We noticed some differences in the 3’-bias values between the samples; so, in order to correct them, we proceeded as described in the paper of Sigurgeirsson et al. [25] with some additional steps. Particularly, even after adjustment, we still noted the association of log2[fold change] values from the log2[avg. transcript length per gene], which indicates the incomplete elimination of bias. For this reason, we linearly approximated this dependency and subtracted the derived straight line to eliminate the slope. A similar procedure was performed to eliminate the dependency of log2[fold change] from log2[avg. CPM].

Read counts data were analyzed by edgeR using TMM normalization and the quasi-likelihood f-test [26]. In our case, we were dealing with huge expression changes affecting thousands of genes. On the one hand, this makes the influence of the 3’-bias negligible, but on the other hand, it creates difficulties with data normalization, which basically assumes that most of the genes should be stable. Moreover, we cannot use reference genes here because their expression can also be altered by 2–3-fold. For this reason, we used the standard TMM normalization and then manually estimated the densities of gene expression distributions (log2[CPM]) and made sure that the TMM normalization was adequate.

### 2.5. Reverse Transcription—Real Time Polymerase Chain Reaction (RT-qPCR)

Total RNA was purified from 1 × 10^6^ cells using High Pure RNA Isolation Kit (Roche). Two micrograms of total RNA from each sample were subjected to reverse transcription using random hexamer primer and RevertAid reverse transcriptase (Thermo Fisher Scientific) according to the manufacturer’s instructions.

Real-time PCR analysis was carried out as previously described [27,28,29] with the primers listed in the Supplementary Table S1.

### 2.6. Polyamine Quantification

Polyamines were quantified by high pressure liquid chromatography (HPLC) with post-column derivatization with o-phthalic aldehyde, as described previously [16,30].

### 2.7. Quantification of Amino Acids by Gas Chromatography-Mass Spectrometry (GC-MS)

Metabolites were extracted from cells grown on 10 cm dish, dried and derivatized with O-methylhydroxylamine (Sigma) and *N*-trimethylsilyl-*N*-methyl trifluoroacetamide (MSTFA) (Supelco, Bellefonte, PA, USA) according to the standard protocol of O.Fiehn [31]. During extraction, nor-valine (Sigma) was added as a standard. The quantification of proline, glutamate, and pyroglutamate was performed by targeted GC–MS on a Chromatec Crystal-5000 gas chromatograph (Chromatec, Yoshkar-Ola, Russia) coupled to a monoquadrupole Chromatec mass-spectrometry detector (MSD). The samples (1 µL) were injected into a 1 mL/min helium flow in a splitless mode, with electron ionization was performed at 20 eV. The metabolites were separated on a HP-5 ms column (30 m × 0.25 mm, 0.25 μm) (Agilent Technologies, Santa-Clara, CA, USA). The oven temperature was kept at 60 °C for 1 min, ramped to 325 °C at 10 °C/min, and held for an additional 10 min at this temperature. The injector, transfer line, and ion source temperature were set to 250 °C, 290 °C, and 230 °C, respectively. After 8 min of solvent delay, the MS data were acquired in full scan mode from *m/z* 50–600. Raw spectra were analyzed using Chromatec Analytic software (version 3.0.0.2). The quantification of amino acids, including glutamate, proline, and pyroglutamate, was performed in SIM mode using the target ion and retention time for each metabolite. These data were obtained by analyzing their standards prepared using the same derivatization protocol, analytical parameters, and instruments. The target ion and retention time of proline were *m/z* 142 and 11.94 min and they were *m/z* 246 and 15.91 min for glutamate; they were *m/z* 156 and 14.82 min for pyroglutamate; and *m/z* 144 and 11.07 min for nor-valine. The concentration of each compound was calculated from their peak intensities using a calibration curve obtained under the same experimental settings. Then, the values were normalized to the signal of nor-valine used as an internal standard and to the total protein concentration in a sample, as determined by the Pierce BCA Protein Assay Kit (Thermo Fischer Scientific, Waltham, MA, USA).

### 2.8. Western Blotting

The cells from 6-well plates were lysed in RIPA buffer, the lysates were applied on 12% SDS-PAAG, and the proteins, resolved by Laemmly electrophoresis, were transferred onto a nitrocellulose membrane (Hybond ECL, GE Healthcare, Little Chalfont, UK), as described previously [32]. After blocking with 5% nonfat milk in phosphate-buffered saline with 0.05% Tween-20 (PBS-T), the membrane was stained with antibodies to PRODH, ASS1, Nqo1, and β-actin as a housekeeping protein. After washing steps, the membranes were stained with HRP-conjugated secondary antibodies to murine IgG, and the bands were visualized with the Standard ECL or ECL Pico detection system (Thermo Fisher Scientific, Rockford, IL, USA) on Chemidoc MP (Bio-Rad, Hercules, CA, USA).

### 2.9. Statistical Analysis

The data in the plots are presented as means ± standard deviation (S.D.). Statistical significance between groups was assessed by analysis of variance (ANOVA) with Tukey’s post hoc test. A *p*-value of 0.05 or lower was considered to be statistically significant. The graphs present *p*-values for the comparison of groups with the differentiated HepaRG cells).

## 3. Results

### 3.1. ROS-Producing Enzymes

First, we assessed the levels of expression of ROS-producing enzymes in Huh7.5 and HepaRG hepatoma cells in normal conditions and during the activation of polyamine catabolism in an RNAseq dataset previously published in [21]. These enzymes included NADPH-oxidases (NOX), cytochrome P450 (CYP), and various catabolic enzymes. The NOX/DUOX family of NADPH-oxidases comprises seven members: NOX1, NOX2, NOX3, NOX4, and NOX5, as well as dual oxidases 1 and 2 (DUOX1, DUOX2) [33]. These transmembrane proteins are capable of transferring electrons across cell membranes, while reducing molecular oxygen to superoxide anion or hydrogen peroxide. We also analyzed the mRNA levels of two more members of the NADPH oxidase multiprotein complex, the activator of the NOX1 isoform, NOXA1, and the cytoplasmic protein NCF2 (neutrophil cytosolic factor 2). Additionally, in terms of ROS-generating enzymes, we evaluated levels of cytochrome CYP 2E1 (participates in lipid peroxidation [34]), XDH (xanthine dehydrogenase/oxidase—a key protein for the degradation of purines, catalyzing the oxidation of hypoxanthine to xanthine and xanthine to urea with the formation of hydrogen peroxide) [35], MAOA (monoamine oxidase A, mitochondrial protein catalyzing the oxidative deamination of amines) [36], and the two isoforms of the endoplasmic reticulum (ER) oxidoreductase Ero1, producing H2O2 during the oxidation of the thiol groups of proteins [12,37].

**Figure 1:**
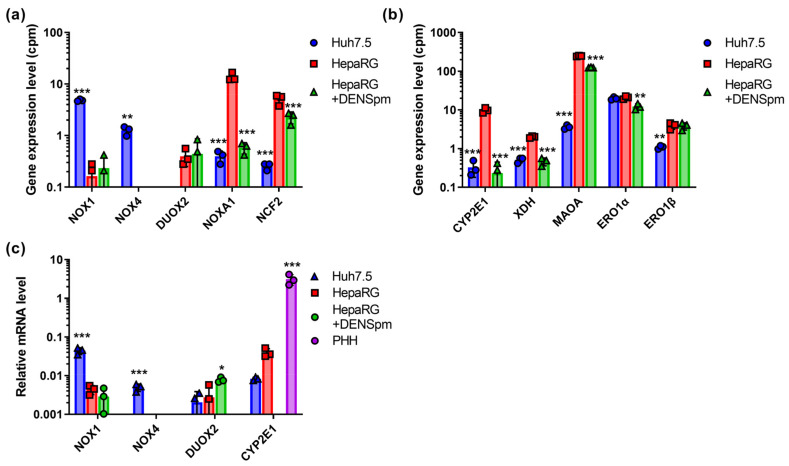
Relative mRNA levels of ROS-producing enzymes in hepatoma Huh7.5 cells, hepatocyte-like HepaRG cells, and HepaRG cells treated with DENSpm, assessed by RNAseq analysis. (**a**) NADPH-oxidases from the NOX/DUOX family. (**b**) Catabolic enzymes. (**c**) Relative levels of the mRNA of ROS-producing enzymes in these cells and primary human hepatocytes (PHH) assessed by RT-qPCR and normalized to the levels of β-glucoronidase (GUS) mRNA. Bars are means ± S.D. Statistical significance was analyzed by ANOVA with Tukey’s post hoc test. Statistical significance is presented for the comparison of cells with the differentiated HepaRG cells. * *p* < 0.05, ** *p* < 0.01, *** *p* < 0.001.

mRNA levels of Nox2, 3, and 5, as well as DUOX1, were below the level of detection, apparently due to the extremely low gene expression of these proteins in liver cells (Figure 1a). The expression levels of Nox1 and Nox4 were higher for the Huh7.5 line, while in HepaRG cells, the levels of their transcription remained very low. These data were verified by reverse-transcription and quantitative PCR (RT-qPCR), using the cDNA of PHH as an additional control. As one can see in Figure 1c, the levels of the transcriptions of NOX1 and NOX4 were indeed elevated in tumor Huh7.5 cells compared to non-tumor HepaRG or PHH. For DUOX2, no statistically significant differences in RT-qPCR tests were observed. However, the levels of its mRNA were close to the limit of detection (Ct values >30). This correlated with our previous observation that the quantification of transcripts that are present in RNAseq data at levels below 3 cpm is difficult when using RT-qPCR.

Ero1α showed no significant differences for both cell lines. The transcripts of all other enzymes were expressed to a significantly stronger level in HepaRG cells (Figure 1). Upon the activation of polyamine catabolism and concomitant cellular dedifferentiation, there was a sharp decrease in the level of NOXA1 and CYP2E1. A similar, although less pronounced, effect was observed for NCF2, XDH, MAOA, and Ero1α. Similar changes in CYP2E1 expression were also observed in RT-qPCR assays. However, the levels of its mRNA were lower than in PHH, which can be explained by the fact that this gene is expressed in mature hepatocytes. In the case of DUOX2 and Ero1β, dedifferentiation had no effect on their expression.

### 3.2. ROS-Scavenging Enzymes

**Figure 2:**
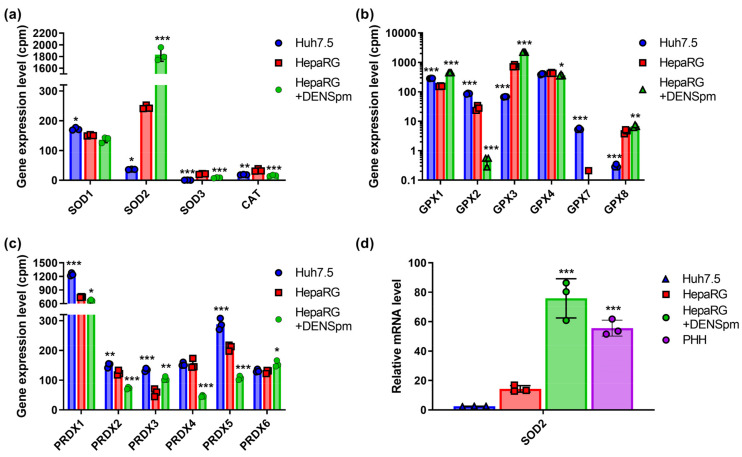
Relative mRNA levels of ROS-scavenging enzymes in hepatoma Huh7.5 cells, hepatocyte-like HepaRG cells, and in HepaRG cells treated with DENSpm, assessed by RNAseq analysis. (**a**) Levels of superoxide dismutases (SOD) and catalase (CAT) mRNA, (**b**) levels of glutathione peroxidases (GPx) mRNA, and (**c**) levels of peroxiredoxins mRNA. (**d**) Relative levels of superoxide dismutase 2 (SOD2) mRNA in these cells and PHH assessed by RT-qPCR and normalized to the levels of GUS mRNA. Bars are means ± S.D. Statistical significance was analyzed by ANOVA with Tukey’s post hoc test. Statistical significance is presented for the comparison of cells with the differentiated HepaRG cells. * *p* < 0.05, ** *p* < 0.01, and *** *p* < 0.001.

Next, we analyzed the levels of the expression of ROS-scavenging enzymes. These included superoxide dismutases (SOD), which are enzymes that catalyze the reaction of the disproportionation of the superoxide radical (O2•−) into molecular oxygen (O2) and hydrogen peroxide (H2O2); catalase (CAT), which decomposes hydrogen peroxide into water and molecular oxygen, is capable of oxidizing low molecular weight alcohols and nitrites in the presence of hydrogen peroxide; and glutathione peroxidases and peroxiredoxins, which catalyze the reduction of organic hydroperoxides to alcohols and hydrogen peroxide to water [38,39,40,41]. The families of superoxide dismutases, glutathione peroxidases, and peroxiredoxins are represented by three, eight, and six proteins, respectively. Catalase has one isoform. It has been shown that in Huh7.5 hepatoma cells and non-tumor HepaRG liver cells, there are no statistically significant differences in the expression levels of SOD1, SOD3, CAT (Figure 2a), GPx4 (Figure 2b), Prdx2, Prdx4, and Prdx6 (Figure 2c). The expression levels of SOD2, GPx3, and GPx8, on the other hand, were much higher in the HepaRG line (Figure 2a,b). The levels of GPx1 and 2 and Prdx1, 3, and 5 in hepatoma cells were increased compared to hepatocyte-like cells, while GPx3 was markedly reduced. Finally, opposite results were obtained in GPx7 and 8—the former was expressed in Huh7.5 cells, while the latter was expressed in HepaRG.

The dedifferentiation of HepaRG cells in response to the activation of polyamine catabolism also changed the expression levels of a number of these enzymes: in the case of SOD2, GPx1, GPx3, and Prdx3, an increase in expression was noted, while for Prdx2, 4, and 5, it decreased markedly, and in the case of GPx2, it dropped below detection level. In addition, a strong increase in the expression of SOD2, GPx1, and GPx3 was observed in response to the activation of polyamine catabolism.

As the most pronounced differences were observed in case of SOD2, the levels of mRNA were also analyzed by RT-qPCR. Indeed, they were higher in the differentiated HepaRG cells compared to Huh7.5 cells, and the activation of polyamine catabolism further increased the levels of mRNA (Figure 2d).

### 3.3. Thoiredoxin and Glutaredoxin Systems

**Figure 3:**
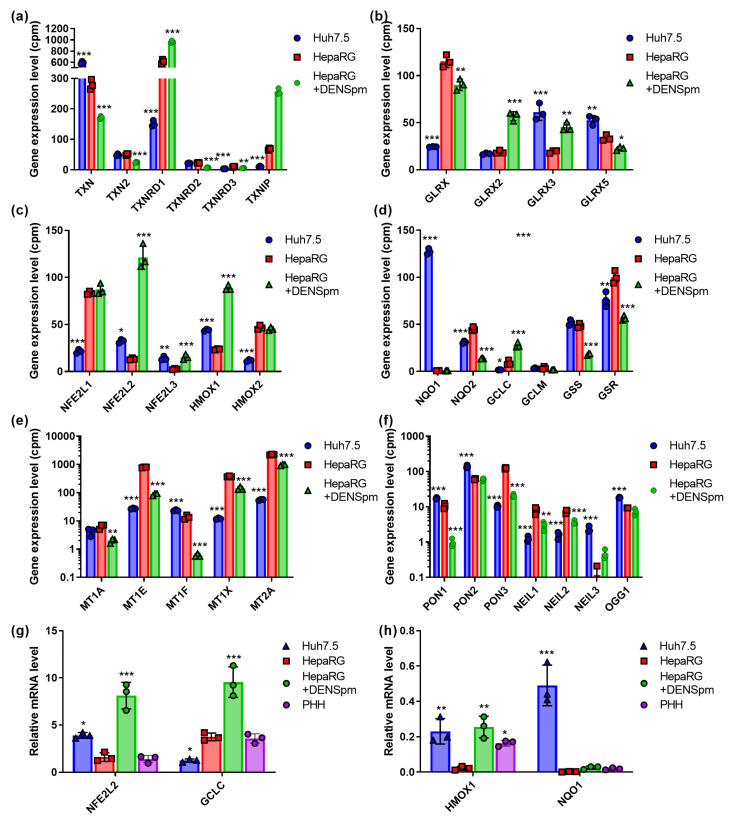
Relative mRNA levels of antioxidant enzymes and proteins that protect DNA and lipids from oxidative damage in hepatoma Huh7.5 cells, hepatocyte-like HepaRG cells, and in HepaRG cells treated with DENSpm, assessed by RNAseq analysis. Gene expression levels of thioredoxins (TRX) and thioredoxin reductases (TRXR) (**a**), glutaredoxins (GLRX) (**b**), Nrf2-dependent enzymes (**c,d**), metallothioneins (MT) (**e**), and paraoxonases (PON) and DNA glycosylases (**f**). (**g,h**) Relative levels of the mRNA of the antioxidant enzymes in these cells and PHH assessed by RT-qPCR and normalized to the levels of GUS mRNA. Bars are means ± S.D. Statistical significance was analyzed by ANOVA with Tukey’s post hoc test. Statistical significance is presented for the comparison of cells to differentiated HepaRG cells. * *p* < 0.05, ** *p* < 0.01, *** *p* < 0.001.

The redox status of a cell is tightly regulated by thioredoxins (TXN) ang glutaredoxins (GLRX) and their reductases. These enzymes restore oxidized peroxiredoxins and glutathione peroxidases [39,42]. RNAseq analysis revealed that hepatocyte-like HepaRG cells show lower levels of the expression of TXN but higher levels of reductase (TXNRD1) and glutaredoxin (GLRX) (Figure 3a,b). The activation of polyamine catabolism further increased these differences. Similar changes were observed for thioredoxin-interacting protein (TXNIP) (Figure 3a), which plays a role in the development of various liver redox-associated pathologies [43].

### 3.4. Nrf2/ARE Cascade

Nuclear factor (erythroid-derived 2)-like factor (NFE2L2, Nrf2) is a transcription factor that controls the expression of various antioxidant and detoxifying enzymes that contain an ARE (antioxidant response element) regulatory sequence in their promoter region [44]. Here, we assessed RNA levels for the following Nrf2-dependent genes: heme oxygenase (HMOX1) and quinone oxidoreductase (NQO1) (both proteins are represented by two isoforms) and catalytic (GCLC) and modifying (GCLM) subunits of glutamate-cysteine ligase, as well as enzymes of biosynthesis of glutathione (glutathione synthetase—GSS; glutathione reductase—GSR). In addition, we quantified the mRNA of Nrf2 as well as that of two other Nrf protein isoforms, Nrf1 and Nrf3 (NFE2L1, NFE2L3), which also affect the transcription of ARE-dependent genes [45,46]. This revealed that Nrf1 and Nrf2 behave in exactly the opposite ways: Nrf1 is more expressed in hepatocyte-like HepaRG cells, whereas the level of Nrf2 is higher in the hepatoma Huh7.5 line (Figure 3c). The activation of polyamine catabolism and concomitant cell dedifferentiation has no noticeable effect on Nrf1 but significantly enhances Nrf2 expression. Nrf3 is generally more similar to Nrf2, although its expression in cells is low and the effect of dedifferentiation is not so pronounced. A similar pattern was seen for the Nrf2-dependent HMOX1 gene. DENSpm also induced the transcription of Nrf2 HMOX1 and GCLC genes (Figure 3c,d). Importantly, for all these genes, mRNA levels in differentiated HepaRG cells were comparable to those in PHH. RT-qPCR verified lower levels of the transcription of GCLC in Huh7.5 compared to Huh7.5 (Figure 3g). Finally, RT-qPCR (Figure 3h) and Western blotting (Figure S1) verified the profoundly higher expression of NQO1 in tumor Huh7.5 cells than is seen in non-tumor HepaRG or PHH cells.

To assess status of the Nrf1 transcription factor, we analyzed the mRNA levels of metallothioneins that are selectively controlled by this protein [45]. In lines with higher levels of Nrf1expression, HepaRG cells demonstrated the enhanced levels of the transcription of MT1E, MT1X, and MT2A (Figure 3e).

### 3.5. Enzymes Protecting DNA from Oxidative Damage

Free radicals can interact with each other. Superoxide anions react with nitric oxide to produce peroxynitrite, which decomposes to form a hydroxyl radical [47]. Hydroxyl radicals impose threat to various biological molecules including DNA, as it is the least stable and most active type of ROS [40]. Here, we analyzed the mRNA levels of enzymes of DNA excision repair, such as 8-oxoguanine glycosylase OGG1, and the three isoforms of NEIL glycosylase (Nei-Like DNA glycosylase), as well as of paraoxonases (PON) 1–3. Hepatocyte-derived PON1, as well as its analogs, associate with lipoproteins and protect them against oxidative damage, thus acting as antioxidant enzymes [48]. PON1, PON2, and OGG1 demonstrated a higher level of expression in Huh7.5 cells compared to HepaRG; for PON3, the situation was reversed (Figure 3f). The activation of polyamine catabolism reduced the expression level of PON1 and PON3 but not PON2 and OGG1. NEIL1 and NEIL2 showed similar expression profiles to those of PON3—they were the predominant forms of the enzyme in hepatocyte-like HepaRG cells and showed a marked decrease in expression upon dedifferentiation (Figure 3d). For NEIL3, the situation was the opposite: expression was higher for Huh7.5 cells, while cell dedifferentiation increased its level.

### 3.6. Polyamine-Metabolizing Enzymes

**Figure 4:**
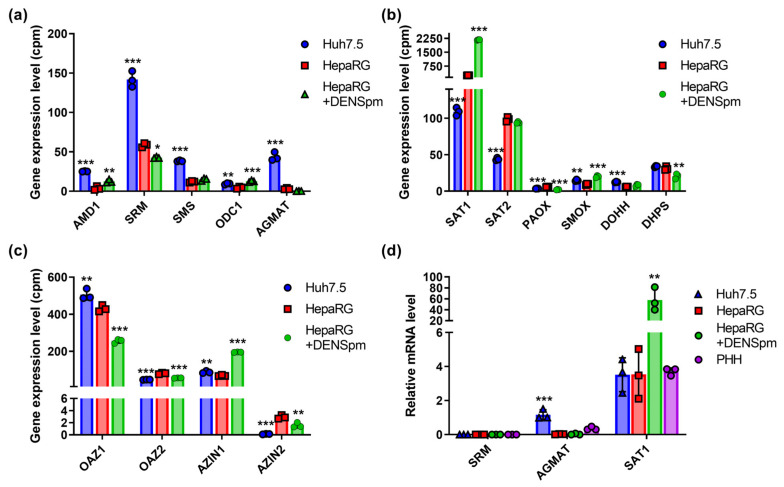
Relative mRNA levels of enzymes of polyamines metabolism in hepatoma Huh7.5 cells, hepatocyte-like HepaRG cells, and HepaRG cells treated with DENSpm, assessed by RNAseq analysis. Levels of the mRNA of polyamine-metabolizing enzymes (**a**), enzymes of urea cycle (**b**), or proline metabolism (**c**). (**d**) Relative levels of mRNA of metabolic genes in these cells and PHH assessed by RT-qPCR and normalized to the levels of GUS mRNA. Bars are means ± S.D. Statistical significance was analyzed by ANOVA using Tukey’s post hoc test. Statistical significance is presented for the comparison of cells to differentiated HepaRG cells. * *p* < 0.05, ** *p* < 0.01, *** *p* < 0.001.

Polyamine biosynthesis is regulated by enzymes encoded by the ODC, spermidine and spermine synthases (SRM and SMS), and AMD genes, while catabolism is regulated by SAT1, SMOX, and acetylpolyamine oxidase (PAOX) [12]. Transcriptome analysis revealed that in Huh7.5 hepatoma cells, the expression of polyamine biosynthesis genes (SRM, SMS, and AMD) was increased, while in hepatocyte-like HepaRG cells, the expression of the SAT1 gene involved in the degradation of spermine and spermidine was increased (Figure 4a,b). Similar changes were found for the SAT2 isoform (Figure 4b) that encodes a functionally inactive protein [49]. It is noteworthy that RT-qPCR failed to verify higher levels of SAT1 mRNA in HepaRG cells compared to Huh7.5 (Figure 4d). At the same time, the level of the mRNA of polyamine oxidases PAOX and SMOX in two cell lines remained very low and differed insignificantly. Similar levels of expression were observed for the isoforms of regulatory ornithine decarboxylase antizyme (OAZ) and the antizyme inhibitor (AZIN) (Figure 4c). The only exception was AZIN2—the minor variant of the protein. The activation of polyamine catabolism decreased levels of OAZ1 and increased levels of AZIN1, presumably to allow the induction of the biosynthetic pathway. One should note that regulation by OAZ1 is likely to be regulated in post-transcriptional step, as functional antizyme is produced upon a polyamine-dependent frameshift during the translation of its mRNA [50] and, probably, during its polyamine-dependent dimerization [51]. In Huh7.5 cells, the mRNA of the agmatinase (AGMAT) was also detected, which was not observed in the non-tumor HepaRG line, as was revealed through both RNAseq and RT-qPCR analysis (Figure 4a,d). The treatment of HepaRG cells with DENSpm and the concomitant activation of SAT1 transcription also significantly increased the level of ODC and SMOX expression. Finally, there were only slight differences in the expression of genes involved in the unique polyamine-dependent post-translational modification of translation initiation factor eIF5a, deoxyhypusine synthase (DHPS), and deoxyhypusine hydroxylase (DOHH) (Figure 4b).

In order to verify that these differences in the transcription of polyamine-metabolizing enzymes affect the metabolic pathway, polyamine levels were determined via the HPLC method described previously [30]. The results, presented in Table 1, clearly show that the elevation of SSAT mRNA levels in HepaRG cells treated with DENSpm result in a decrease in spermine and spermidine levels. Moreover, the increased transcription of most enzymes of polyamine biosynthesis (ODC, AMD1, SRM, and SMS) in Huh7.5 cells compared to HepaRG is accompanied by the accumulation of both spermine and spermidine. It is also worth noting that significantly lower levels of ADM1 and SRM in the HepaRG cells allowed the detection of putrescine, which is readily converted into spermidine in Huh7.5 cells.

### 3.7. Urea Cycle and Proline Metabolism

**Figure 5:**
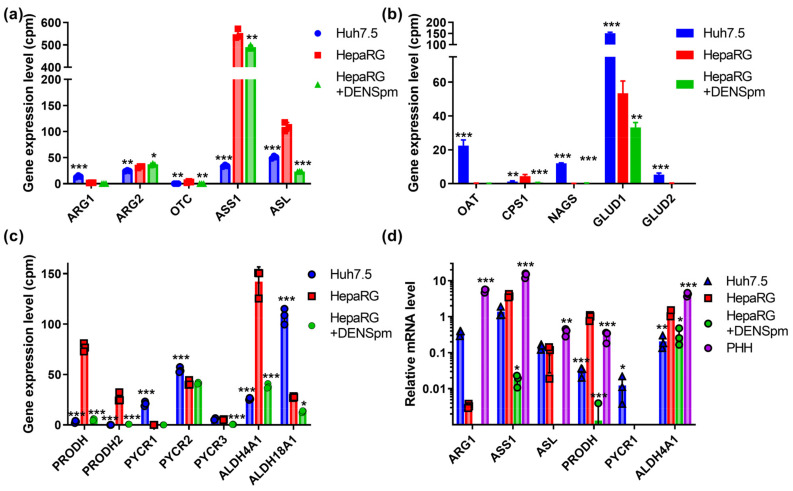
Relative mRNA levels of urea cycle and proline metabolizing enzymes in hepatoma Huh7.5 cells, hepatocyte-like HepaRG cells, and HepaRG cells treated with DENSpm, assessed by RNAseq analysis. Levels of the mRNA of the encoding enzymes of the urea cycle (**a**) or of the proline metabolism (**b**,**c**). (**d**) Relative levels of mRNA of metabolic genes in these cells and PHH assessed by RT-qPCR and normalized to the levels of GUS mRNA. Bars are means ± S.D. Statistical significance was analyzed by ANOVA with Tukey’s post hoc test. Statistical significance is presented for the comparison of cells to differentiated HepaRG cells. * *p* < 0.05, ** *p* < 0.01, *** *p* < 0.001.

Next, we assessed the mRNA levels of enzymes of the urea cycle and proline metabolism, since the polyamine precursor putrescine is synthesized from a non-proteinogenic amino acid ornithine of this cycle, which is also linked to Pro/Glu metabolic system [12,52]. The results, presented in Figure 5a–c, show that in hepatocyte-like HepaRG cells, the level of the transcript encoding argininosuccinate synthase (ASS1) is fifteen-fold higher than in Huh7.5 cells, and the mRNA of the argininosuccinate lyase gene (ASL), which synthesizes arginine, is two times higher. Similar ratios were observed for carbamoylphosphate synthase 1 (CPS1). On the other hand, in hepatoma cells, the expression levels enzymes involved in ornithine biosynthesis are increased, namely arginase 1, ornithine aminotransferase (OAT), and N-acetylglutamate synthase (NAGS), as well as enzymes that convert glutamate to α-ketoglutarate (GLUD1 and GLUD2) and utilize ammonium. The activation of the catabolism of biogenic polyamines significantly affected only the level of ASL gene expression. The verification of these results by RT-qPCR supported the conclusion concerning the differences in ARG1 and ASS1 expression between Huh7.5 and HepaRG cells, as well as the decreased transcription of ARG1 and ASL in response to the activation of polyamine catabolism (Figure 5d). Additionally, ASS1 levels were increased in HepaRG cells compared to Huh7.5 cells, according to immunoblot analysis (Figure S1).

Compared to the hepatoma Huh7.5 cell line, HepaRG cells have a number of differences in proline metabolism. For example, the expression of enzymes producing delta-1-pyrroline-3-hydroxy-5-carboxylate (P5C) in HepaRG cells is significantly higher than in the Huh7.5 cell line (Figure 5c). These enzymes include proline dehydrogenase 1 (PRODH), which catabolizes proline into P5C, and proline dehydrogenase 2 (PRODH2), which, in addition to proline, also catabolizes 4-hydroxyproline [53]. The expression of PYCR2, responsible for the synthesis of proline in mitochondria from P5C, in HepaRG cells was lower, while the expression of PYCR3 that synthesized proline in the cytoplasm did not differ between HepaRG and Huh7.5. The transcription of the PYCR1 gene was observed only in the Huh7.5 line, both in RNAseq and RT-qPCR experiments (Figure 5c,d). The Huh7.5 cells were also characterized by the conversion of glutamate to P5C due to the increased transcription of the ALDH18A1 gene. In HepaRGs, proline catabolism to glutamate is significantly enhanced, as ALDH4A1, PRODH1 and PRODH2 mRNAs were present at higher levels; again, this was revealed through transcriptomic, RT-qPCR, and Western blot analysis (Figure 5 and Figure S1). During the dedifferentiation of HepaRG, there was a decrease in the transcription of all the genes of the proline cycle and P5C synthesis/catabolism, with the exception of PYCR2.

Additional verification was performed through the GC–MS analysis of proteinogenic and nonproteinogenic amino acids. Unfortunately, this method was found to be inapplicable for the quantification of urea cycle metabolites, such as arginine, citrulline, and arginonosuccinate, on trimethylsilylation (required derivatization) are converted into ornithine [54]. So, verification was based on the quantification of glutamate, proline, and pyroglutamate, as the latter spontaneously converted into pyrrolidine-5-carboxylate (Scheme 1). The ratios between these metabolites depends on the expression of PRODH and PYCR on one hand and ALDH4A1 and ALHD18A1 on the other. The higher expression of proline-catabolizing enzyme (PRODH) and the lower expression of the proline-biosynthetic enzyme (PYCR1) in HepaRG compared to the Huh7.5 cells was indeed accompanied by a dramatic decrease in the intracellular level of proline and a shift in proline/pyroglutamate ratio (Table 2). Moreover, the increased expression of ALDH4A1 and decreased ALDH18A1 was associated with an increased glutamate/pyroglutamate ratio (3.25 ± 0.28 vs. 2.32 ± 0.07).) However, treatment with DENSpm had no effect on concentrations of these amino acids, although it affected the levels of asparagine/aspartate (data not shown). So, the changes in proline, glutamate, and pyroglutamate (pyrrolidine-5-carboxylate) could be more dependent on PYCR and ALDH18A1 than on PRODH and ALDH4A1. However, overall, the changes support the concept that the levels of the transcription of these metabolic enzymes correlate with their functionalities in cells.

### 3.8. Calcium Transporters and Their Regulatory Proteins

**Figure 6:**
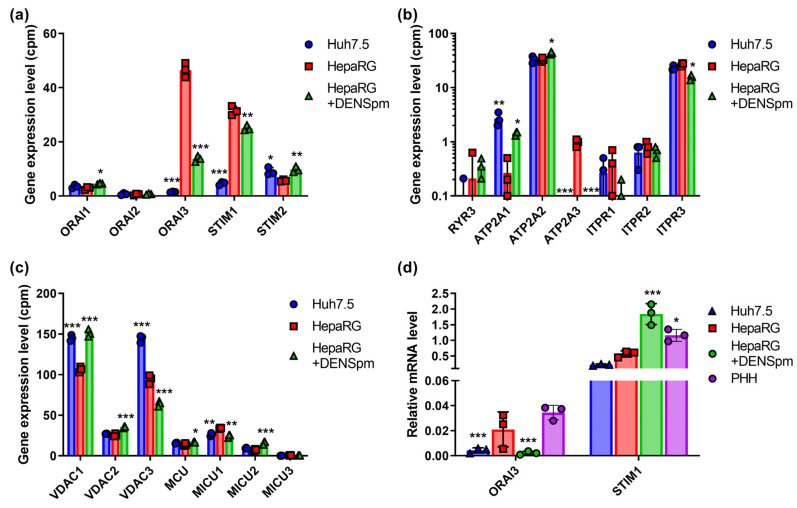
Relative mRNA levels of the genes of calcium homeostasis in hepatoma Huh7.5 cells, hepatocyte-like HepaRG cells, and HepaRG cells treated with DENSpm, assessed by RNAseq analysis. Levels of mRNA of plasmatic calcium transporters and auxiliary proteins (**a**), ER calcium pumps (**b**), or of mitochondrial transporters (**c**). (**d**) Relative levels of ORAI3 and STIM1 mRNA in these cells and PHH assessed by RT-qPCR and normalized to the levels of GUS mRNA. Bars are means ± S.D. Statistical significance was analyzed by ANOVA using Tukey’s post hoc test. Statistical significance is presented for the comparison of cells to the differentiated HepaRG cells. * *p* < 0.05, ** *p* < 0.01, *** *p* < 0.001.

The final aim of the work was to analyze the expression of various calcium ion transporters on the plasma membrane, endoplasmic reticulum, and mitochondria, as calcium homeostasis is tightly linked with redox status of a cell. The results are shown in Figure 6. One can see that Huh7.5 and HepaRG lines differ both in the mRNA levels of the ORAI calcium channel proteins and in the mRNA levels of their STIM adapter proteins (Figure 6a). In hepatoma cells, ORAI1 and STIM2 adapter protein were the predominant forms, while in HepaRG cells, transcription of ORAI3 and STIM1 was much higher, as revealed in RNAseq dataset and by real-time PCR (Figure 6a,d). Activation of polyamine catabolism triggered a significant drop in the level of ORAI3 mRNA.

Among the three types of inositol trisphosphate (ITP3) receptors, ITPR3 was the most expressed isoform in both cell lines, with minor differences in transcription levels between Huh7.5 and HepaRG (Figure 6b). The expression of the ITPR1 and ITPR2 genes was close to the threshold of detection. Additionally, the mRNA of ryanodine receptor RYR3, which is responsible for the alternative release of Ca^2+^ into the cytoplasm from the ER, was absent in both cell lines. Calcium homeostasis in mitochondria between Huh7.5 hepatoma cells and HepaRG cells did not differ. The ratio of the expression of the three isoforms of voltage-dependent anion-selective channel proteins (VDAC), located on the outer membrane of mitochondria, was almost the same—the expression of VDAC1 and VDAC3 predominated (Figure 6c). During HepaRG dedifferentiation, a moderate decrease in the transcription of the VDAC3 gene and an increase in the VDAC1 transcript were observed. The levels of the transcription of the calcium uniporter protein on the inner mitochondrial membrane (MCU) between Huh7.5 and HepaRG cell lines were also similar, and MICU1 was the main isoform in both cell lines. Among the three paralogs of the calcium ATPase SERCA (ATP2A) responsible for the import of calcium from the cytoplasm into the ER, only SERCA2 was expressed. SERCA2 transcript levels were similar between Huh7.5 and HepaRG cell lines.

## 4. Discussion

NOX/DUOX NADPH-oxidases are one of the major sources of ROS in a cell during various pathologies [33]. This family of enzymes includes seven members that have distinct expression patterns. Previously, we and other reported that NOX1 and NOX4 were induced by the hepatitis C virus (HCV) in Huh7.5 cells, which contributes to virus-associated oxidative stress [32,55,56,57]. However, in this paper, we show that in uninfected Huh7.5 cells, only NOX1 and NOX4 were detected with low expression levels. Moreover, in non-tumor hepatocyte, such as HepaRG cells, NOX1 and NOX4 are even lower (at least by an order of magnitude); however, in these, DUOX2 is readily detected. So, the assessment of the input of the various members of the NOX/DUOX family in ROS production and the development of associated liver pathology can lead to opposite conclusions, depending on the liver cell line used.

Superoxide anions, a major type of ROS, are neutralized by superoxide dismutases. Their family comprises three isoforms: cytoplasmic SOD1, mitochondrial SOD2, and extracellular SOD3 [38]. It is noteworthy that SOD2 also plays a role in cell cycle regulation by changing the ratio between superoxide anion and hydrogen peroxide in mitochondria [58]. The higher level of its expression in HepaRG cells shown here, which correlates with data that HepaRG^diff^ is present in the G0/G1 phase of the cell cycle [59] and that its expression reaches a maximum in the G1 phase [60]. An additional increase in SOD2 expression during DENSpm treatment may point to either the enhanced production of superoxide anions in mitochondria during enhanced polyamine catabolism or to the dependence of SOD2 expression on the (de)differentiation of hepatocytes.

Glutathione peroxidases (which have eight isoforms) and peroxiredoxins (represented by six isoforms), found in various cellular compartments, efficiently utilize intracellular hydrogen peroxide, organic peroxides, and peroxide products and lipid oxidation [39,41]. In both types of analyzed liver cells, GPx5 and 6 were not detected, while GPX1, GPX4, and peroxiredoxins, with the exception of isoforms 3 and 4, were expressed at similar levels. Non-tumor HepaRG cells exhibited a much higher expression of GPX8 and a much lower expression of GPx7. Notably, both of these enzymes are responsible for scavenging hydrogen peroxide in the endoplasmic reticulum [39]. Thus, we may assume that both types of cells should have a similar ability to prevent H_2_O_2_ leakage from the ER. The HepaRG cells also showed a much higher expression of GPx4, correlating with literature data regarding GPx4 functioning as a tumor suppressor [61]. The activation of polyamine catabolism by DENSpm treatment and concomitant enhanced ROS production led to the marked inhibition of the expression of GPx2 and Prdx4 and the enhancement of GPx1, GPx3, and Prdx3 gene transcription. It is tempting to speculate that the upregulation of the expression of these predominantly cytoplasmic peroxidases [62] could point to the enhancement of H_2_O_2_ production in cytosol by SSAT. At the same time, we should mention that the downregulation of GPx2 under the dedifferentiation of HepaRG cells, i.e., towards liver progenitor cells [21], contradicts the fact that GPx2 is preferably expressed in stem and progenitor cells [63]. Finally, changes in the expression of ER-residing Prdx4, in response to enhanced polyamine catabolism, provide an additional link between polyamines and ER homeostasis [64,65]. Indeed, DENSpm has previously been shown to alter ribosome interaction with the ER, affecting one of its major functions [62].

Compared to hepatoma Huh7.5 cells, HepaRGs demonstrated lower levels of expression of the Nrf2 transcription factor, which, however, was not accompanied by decreased levels of the transcription of the Nrf2/dependent enzymes. At the same time, the activation of polyamine catabolism and concomitant oxidative stress enhanced the expression of Nrf2 and GCLC, which correlates with the overproduction of the hydrogen peroxide [66]. However, again, several other Nrf2/dependent genes did not change their transcription rates. Interestingly, HepaRG cells exhibited the higher expression of Nrf1—a homologue of Nrf2 that also controls the transcription of ARE-dependent genes [67]. As Nrf1 selectively induces the expression of metallothioneins [45], it is not surprising that the levels of MT1E, MT1X, and MT2A mRNAs were also higher than in Huh7.5 cells.

The comparison of proliferative hepatoma Huh7.5 cells with non-proliferative differentiated non-tumor HepaRG cells revealed significantly higher levels of the mRNAs of polyamine biosynthesis genes (AMD, ODC, SRM, SMS) and lower levels of SAT1, which encodes the main catabolic enzyme with non-constitutive expression. This perfectly matched a previous finding of the lower levels of spermine and spermidine in HepaRG cells [21]. In our opinion, it is very important to stress that the lowest level of transcripts of various polyamine-metabolizing genes was noted for PAOX, which encodes acetylpolyamine oxidase. This enzyme is considered responsible for the conversion of SSAT products, acetylspermine and acetylspermidine, into spermidine and putrescine, respectively. However, as the levels of its mRNA were close to the limit of detection, this calls into question its real contribution to polyamine catabolism. Moreover, the different intracellular localization of SAT1 and PAOX (cytoplasmic and peroxisomal, respectively [62,68]) also suggests that PAOX’s role in metabolism should be re-examined. At the same time, DENSpm could drive the conversion of spermine into spermidine through the induction of spermine oxidase, which is also localized in cytoplasm [69].

Polyamine precursor ornithine is a metabolite of the urea cycle. The main differences in urea cycle gene expression between the Huh7.5 hepatoma cell line and HepaRG hepatocyte-like cells are differences in arginine synthesis. The expression levels of arginine succinate synthase (ASS1) and arginine succinate lyase (ASL) genes in HepaRG cells are significantly higher than in Huh7.5, which should affect the efficiency of arginine synthesis. However, it may also suggest that in the HepaRG cells maintained in William’s medium, arginine may be converted not into ornithine but into argininosuccinate, as shown recently in DMEM-F12 medium in non-liver cells [70]. The latter could be due to non-physiologically high levels of arginine in such media (reviewed in [71]). Another important difference to the Huh7.5 cancer line is the more efficient transcription of the OAT gene that links ornithine and urea cycles with the proline metabolic pathway [72]. In contrast, the expression of this gene was undetectable in the HepaRG cell line. This may indicate that in non-tumor liver cells, proline/glutamine metabolism is not linked to urea cycle/polyamine metabolism and does not contribute to polyamine biosynthesis.

Notable differences between the Huh7.5 and HepaRG cell lines in the expression of proline metabolism genes were also observed. In particular, the transcription of the PYCR1 gene was only observed in the Huh7.5 hepatocyte cancer line, indicating it to be a possible marker of hepatoma cells. In Huh7.5 hepatoma cells, the expression of proteins synthesizing proline from P5C (PYCR1, PYCR2, PYCR3) prevails over proline-utilizing proteins (PRODH and PRODH2) and the coordinated action of PYCR3, PRODH, and PRODH2 proteins. P5C, being exported into the cytoplasm, is converted into proline, resulting in proline being transferred back to the mitochondria, where it was again oxidized into P5C by PRODH and PRODH2 [52]. Hence, a closed cycle leads to an increase in the concentration of NADP^+^ in the cytoplasm and the generation of ROS in mitochondria [73]. When HepaRG cells are dedifferentiated using DENSpm in cells, this cycle is disrupted: the expression levels of PRODH and PRODH2 decrease along with PYCR3, while the PYCR2 expression remains unchanged—the equilibrium shifts towards an increase in proline synthesis. The hypothesis regarding the importance of proline for hepatoma cells is also supported by the fact that the Huh7.5 cell line is characterized by the conversion of glutamate and ornithine to proline due to the increased transcription of the delta-1-pyrroline-5-carboxylate synthase genes (ALDH18A1) and ornithine aminotransferase (OAT) generating P5C in mitochondria. The latter is converted into proline by pyrroline-5-carboxylate reductases 1, 2, and 3. In the hepatocyte-like non-cancerous cell line HepaRG, the reverse process of proline catabolism to glutamate is significantly enhanced due to the increased transcription of the genes for delta-1-pyrroline-5-carboxylate dehydrogenase ALDH4A1 and proline oxidases PRODH and PRODH2, as well as due to the decreased expression of PYCR.

Huh7.5 cells also efficiently express the AGMAT1 gene encoding agmatinase. Agmatinase had previously been identified in humans, and an increase in its transcription during infection with hepatitis B virus was reported [74,75]. In mammals, the arginine decarboxylase gene responsible for the synthesis of agmatine from arginine has not been identified; therefore, agmatinase in Huh7.5 cells can only utilize agmatine from the gastrointestinal tract. This may indicate the role of agmatine as an alternative precursor of putrescine in tumor cells.

Huh7.5 and HepaRG cell lines differed in the expression of ORAI calcium channel proteins. In hepatocyte-like HepaRG cells, ORAI3 was the predominant form of the transporter. A change in the ORAI1/ORAI3 expression ratio affects the sensitivity of cells to the effects of ROS on calcium import from the intercellular medium [76], suggesting that HepaRG is less sensitive to the effect of ROS on this process. HepaRG cells also had much higher levels of STIM1, which is a linker protein between ER and the plasma membrane. Therefore, it can be assumed that in non-tumor cells, the number of such contacts is higher, and thus, the influx of extracellular calcium ions is more efficient [77]. It is worth noting the absence of the mRNA of ryanodine receptor proteins (RYR3) in both cell lines. Apparently, they do not play a role in liver cells. The identical ratio between VDAC1, VDAC2, and VDAC3 mRNA suggests that for both cell lines, there is no difference in the structure and regulation of VDAC calcium channels. A similar situation is observed for the calcium uniporter and its regulation based on the identical ratio of MCU, MICU1, and MICU2 transcripts.

## 5. Conclusions

The data, presented in the current manuscript, clearly show that non-tumor and tumor liver cells have different profiles of the expression of ROS-producing and metabolizing enzymes. This should lead to profound changes between biosynthetic and catabolic pathways, as well as in the fluxes of metabolites between the urea cycle/polyamine pathway on one hand and the proline/glutamate metabolic system on the other.

## Figures and Tables

**Table 1:** Polyamine levels in cell lines.

Cell Line	Polyamine (pmol/ng DNA)		
	Putrescine	Spermidine	Spermine
Huh7.5	N.D.	18.4 ± 6.4	65.5 ± 17.0
HepaRG	0.9 ± 0.3	5.9 ± 1.2	8.4 ± 0.7
HepaRG + DENSpm	N.D.	3.4 ± 0.4	2.3 ± 0.4

**Table 2:** Amino acid levels in cell lines.

Cell Line	Polyamine (nmol/mg Protein)		
	Proline	Pyroglutamate	Glutamate
Huh7.5	34.5 ± 10.6	27.3 ± 6.0	89.3 ± 25.4
HepaRG	1.52 ± 0.03	10.8 ± 0.4	25.1 ± 1.5
HepaRG + DENSpm	1.47 ± 0.09	10.8 ± 0.7	25.6 ± 2.7

## Data Availability

Raw FASTQ Illumina sequence data have been deposited at the Sequence Read Archive (SRA) under the accession number PRJNA494568. The dataset includes 12 records (from SAMN10172596 to SAMN10172607).

## Supplementary Materials

The following supporting information can be downloaded at: https://www.mdpi.com/article/10.3390/biom13040714/s1. Table S1: Oligonucleotides used in real-time PCR; Figure S1: Expression of NAD(P)H:quinone oxidoreductase (Nqo1), proline dehydrogenase (PRODH) and argininosuccinate synthase (ASS1) in hepatoma Huh7.5 cells, hepatocyte-like HepaRG cells and in HepaRG cells treated with DENSpm, assessed by western blot analysis.
