# Supplementary material for: Transcriptome Analysis of Redox Systems and Polyamine Metabolic Pathway in Hepatoma and Non-Tumor Hepatocyte-like Cells
Source: Biomolecules. 2023 Apr 21;13(4):714. doi: 10.3390/biom13040714 (PMC10136275; doi:10.3390/biom13040714)
Supplement: Supplementary file 1 [file biomolecules-13-00714-s001.zip › biomolecules-2212868-supplementary.pdf]

SUPPLEMENTARY DATA

Transcriptome Analysis of Redox Systems and Polyamine Metabolic Pathway in Hepatoma and Non-Tumor Hepatocyte-like Cells

Olga N. Ivanova [1], George S. Krasnov [1], Anastasiya V. Snezhkina [1], Anna V. Kudryavtseva [1], Vyacheslav S. Fedorov [1], Natalia F. Zakirova [1], Michail V. Golikov [1], Sergey N. Kochetkov [1], Birke Bartosch [2], Vladimir T. Valuev-Elliston [1] and Alexander V. Ivanov [1,*]

[1] Engelhardt Institute of Molecular Biology, Russian Academy of Sciences, 119991 Moscow, Russia
[2] Lyon Cancer Research Center, Université Claude Bernard Lyon 1, INSERM U1052, CNRS 5286, 69008 Lyon, France
* Correspondence: aivanov@yandex.ru

Table S1. Oligonucleotides used in real-time PCR.

Gene	Orientation	Sequence (5′-3′)
NOX1	Sense	CAATCTCTCCTGGAATGGCATCCT
	Antisense	CCTGCTGCTCGGATATGAATGGAGAA
NOX4	Sense	CTGGTGAATGCCCTCAACTT
	Antisense	GGCCAGGAACAGTTGTGAAG
DUOX2	Sense	TGGTCATTCTCCTCCACCTC
	Antisense	GCTCCCCAGAGGATAAGTC
CYP2E1	Sense	TTTAAGCCAGAACACTTCC
	Antisense	GCACACAACAAAAGAAACA
SOD2	Sense	GCTGACGGCTGCATCTGTTGGT
	Antisense	AGGCCTGTTGTTCCTTGCAGTGG
NFE2L2	Sense	TACTCCCAGGTTGCCCACA
	Antisense	CATCTACAAACGGGAATGTCTGC
HMOX1	Sense	CCAGCAACAAAGTGCAAGATTC
	Antisense	TCACATGGCATAAAGCCCTACAG
NQO1	Sense	CCGTGGATCCCTTGCAGAGA
	Antisense	AGGACCCTTCCGGAGTAAGA
GCLC	Sense	GGATTTGGAAATGGGCAATTG
	Antisense	CTCAGATATACTGCAGGCTTGGAA
SRM	Sense	CCCGCCGAAAGTCTCTTCAA
	Antisense	GGAAGTTCGTGCTCGGGTTC
AGMAT	Sense	ACATAGCATCTCAAACAGCAGGTTAG
	Antisense	AAGTTTCACCACCGTATGATCTTTC
SAT1	Sense	ATCTAAGCCAGGTTGCAATGA
	Antisense	GCACTCCTCACTCCTCTGTTG
ARG1	Sense	TGATGTGAAGGATTATGGGGac
	Antisense	TGGTTGTCAGTGGAGTGTTG
ASS1	Sense	CAACACCCCTGACATTCTCG
	Antisense	ACTTTCCCTTCCACTCGCTC
ASL	Sense	TCCCAGCACCTACAACAAAGA
	Antisense	TAGTCCCACACGCAGATCACG
ALDH4A1	Sense	CCATCTCGCCCTTTAACTTCAC
	Antisense	ACTGGGCTTCCATAGGACCA

P5CR1	Sense	ACACCCCACAACAAGGAGAC
	Antisense	CTGGAGTGTTGGTCATGCAG
PRODH1	Sense	CAACGCCATGTACCACAGGT
	Antisense	ATGCCTAGCAGCTGTCCAAA
ORAI3	Sense	GTACCGGGAGTTCGTGCA
	Antisense	GGTACTCGTGGTCACTCT
STIM1	Sense	CAGAGTCTGCATGACCTTCA
	Antisense	GCTTCCTGCTTAGCAAGGTT
GUS	Sense	CGTGGTTGGAGAGCTCATTTGGAA
	Antisense	ATTCCCCAGCACTCTCGTCGGT

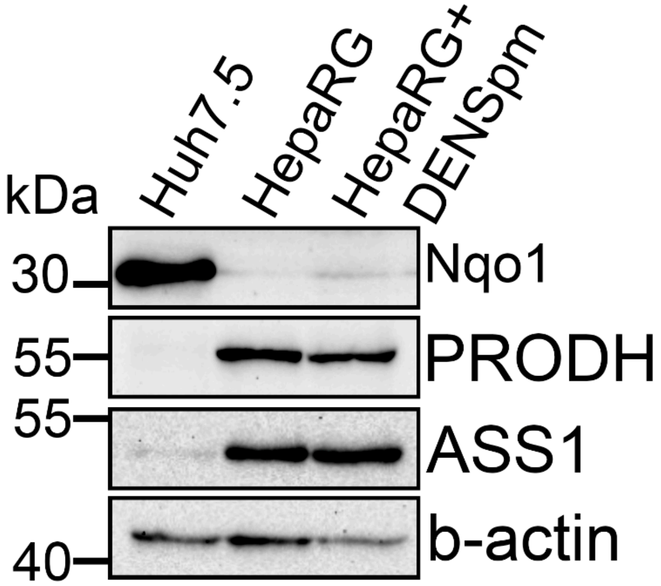

Figure S1. Expression of NAD(P)H:quinone oxidoreductase (Nqo1), proline dehydrogenase (PRODH) and argininosuccinate synthase (ASS1) in hepatoma Huh7.5 cells, hepatocyte-like HepaRG cells and in HepaRG cells treated with DENSpm, assessed by western blot analysis. As a housekeeping gene, β-actin was chosen.
